# Tricuspid annular plane systolic excursion is reflective of biventricular function in critically ill patients

**DOI:** 10.1186/2036-7902-6-S1-A14

**Published:** 2014-01-31

**Authors:** Ratender Singh, Sudeep Kumar, Sreevatsa Nadig, Arvind Baronia

**Affiliations:** 1 SGPGIMS, Department of Critical Care Medicine, Lucknow, India

## Background

Tricuspid annular plane systolic excursion (TAPSE) is an easily measureable,fast and reproducible echocardiographic parameter which gives relevant information about biventricular cardiac function.

## Objective

To explore the utility of tricuspid annular plane systolic excursion (TAPSE) for biventricular function assessment in critically ill ICU patients at admission.

## Design

Prospective observational study.

## Setting

A 12-bed medical-surgical critical care unit.

## Patients and methods

Hundred and one patients admitted to the ICU for sepsis, septic shock, acute cardio respiratory failure or other organ supportivecare. A trained cardiologist conducted transthoracic echocardiography within 48 hours of admission to ICU. Echocardiographic parameters of both right and left heart along with demographic, prognosis, hemodynamic, ventilator, and laboratory parameters wererecorded.

## Results

TAPSE, right ventricle (RV) fractional area change (FAC), the left ventricular ejection fraction (LVEF), were measured using Doppler echocardiography. Mean age and TAPSE of patients was 41.54 ± 16.07 years and 23.09 ± 5.534 mm respectively. Positive correlation of TAPSE was observed with pulsed doppler peak velocity at annulus (right) (N = 95; r = 0.432; p = 0.000), RVFAC (N = 94; r = 0.397; p = 0.000), pulsed doppler peak velocity at annulus (left) (N = 98; r = 0.287; p = 0.004), LVEF(N = 97; r = 0.248; p = 0.014). Also observed was a negative correlation was with tissue doppler myocardial performance index (left) (N = 74; r = -0.226; p = 0.05). On logistic regression analysis TAPSE showed a significant impact on both RVFAC (p = 0.009; Exp (B) = 11.329; 95% C.I = 1.832-70.079) and LVEF (p = 0.040; Exp (B) = 14.154; 95% C.I = 1.127-177.825).

## Conclusions

TAPSE is reflective of biventricular function at baseline in critically ill ICU patients. Its potential as a hemodynamic monitoring tool needs further exploration in critical illness.

